# An analysis of the population of *Cryptococcus neoformans* strains isolated from animals in Poland, in the years 2015–2019

**DOI:** 10.1038/s41598-021-86169-3

**Published:** 2021-03-23

**Authors:** Magdalena Florek, Urszula Nawrot, Agnieszka Korzeniowska-Kowal, Katarzyna Włodarczyk, Anna Wzorek, Anna Woźniak-Biel, Magdalena Brzozowska, Józef Galli, Anna Bogucka, Jarosław Król

**Affiliations:** 1 Department of Pathology, The Faculty of Veterinary Medicine, Wrocław University of Environmental and Life Sciences, Norwida 31, 50-375 Wrocław, Poland; 2 Department of Pharmaceutical Microbiology and Parasitology, Wrocław Medical University, Borowska 211a, 50-556 Wrocław, Poland; 3 Department of Immunology of Infectious Diseases, Hirszfeld Institute of Immunology and Experimental Therapy, Polish Academy of Sciences, Weigla 12, 53-114 Wrocław, Poland; 4 Department of Epizootiology and Clinic of Birds and Exotic Animals, The Faculty of Veterinary Medicine, Wrocław University of Environmental and Life Sciences, pl. Grunwaldzki 45, 50-366, Wrocław, Poland; 5 Referral Animal Hospital Strömsholm, Djursjukhusvägen 11, 73494 Strömsholm, Sweden; 6 Veterinary Laboratory Vetlab, Wodzisławska 6, 52-017 Wrocław, Poland

**Keywords:** Microbiology, Molecular biology, Diseases

## Abstract

Fungi belonging to the *Cryptococcus neoformans/C. gattii* species complex (CNGSC) are pathogens causing severe infections in humans and animals, that for humans may result in a mortality rate ranging up to 70%. The CNGSC is divided into eight major molecular types, that may differ in their virulence and susceptibility. In order to fully understand the epidemiology of cryptococcosis, it is important to study the world distribution and population structure of these pathogens. The present study is the first presenting a population of strains isolated in Poland and one of the few using a multi-species animal group as a source of the specimen. The pathogen was present in 2.375% of the tested animals. The *URA*5-RFLP and MALDI-TOF MS analyses have revealed that the population consisted exclusively of *C. neoformans* strains, with a predominance of major molecular type VNIV (*C. neoformans* var. *neoformans*). The MALDI-TOF MS was used to perform the CNGSC strains identification on both the species and sub-species level. Despite the fact that the animals providing the specimens were not treated with 5-fluorocytosine, around 10% of the tested population presented MIC values exceeding 64 mg/L, indicating the existence of the 5-fluorocytosine-resistant strains in the environment.

## Introduction

Fungi belonging to the *Cryptococcus neoformans/C. gattii* species complex (CNGSC) are pathogens associated with severe infections in humans and in numerous species of animals. Whereas precise data concerning the prevalence of cryptococcosis in animals is not available, it is assumed that in humans more than 220,000 cases of this infection occur annually worldwide, resulting in up to 180,000 deaths^1^.

The described pathogen complex can be divided into eight major molecular types (MMT), namely: VNI, VNII and VNB (all representing *Cryptococcus neoformans* var. *grubii*, serotype A), VNIV (*C. neoformans* var. *neoformans*, serotype D) and VNIII (the hybrid of these two varieties, serotype AD) as well as VGI, VGII, VGIII and VGIV (*C. gattii*, serotype B or C)^2^. It was proposed that within the complex, seven species should be recognised^3^. But since the discussion concerning classification of cryptococci belonging to the group did not reach a consensus among the scientific community, in this paper the nomenclature with regard to a two-species scheme was adopted^4^.

The CNGSC has a bipolar mating system (consisting of one mating-type locus *MAT*), with two different mating types (*MAT*a and *MAT*α) and can readily mate both bisexually and unisexually^5,6^.

It was observed, that the MMTs belonging to the complex can differ with their virulence and susceptibility to antifungals^7,8^ and additionally, as a consequence, they may influence the disease outcome. Geographical distribution of the species belonging to the complex is also various. Whereas *C. neoformans* is present worldwide, *C. gattii* was regarded as associated exclusively with tropical and subtropical zones^9–11^. Since the end of the twentieth century, however, the latter has been detected in countries characterised by a temperate climate^12,13^. As a result of the above-mentioned differences among MMTs and the distribution change, examination of the current population structure and geographic range of the CNGSC is an integral part of epidemiological studies considering the pathogen infections.

Cryptococcosis is an infection that is acquired as a result of inhalation of spores or dehydrated yeast cells from environmental sources such as plant materials or soil as well as bird excreta^14,15^. Various studies were undertaken around the world in order to establish the relationship between cryptococcosis occurrence in humans (as well as in animals) and the presence of the pathogen in the environment^16,17^. In Europe, environmental studies were performed mainly in its Southern and Western regions^15^. Still, data concerning epidemiology of infections with the CNGSC in Central and Eastern parts of Europe is scarce or unavailable^15,18,19^.

Given a closer relationship with plants and soil, exposure to CNGSC is more probable in animals than in humans^20,21^. It was suggested that in companion animals only, prevalence of cryptococcosis appears to be comparable to or even greater than that observed in humans^22–24^. Aside from clinical, other forms of *Cryptococcus* occurrence were observed in animals like subclinical infection and persistent or transient colonization as well as contamination of particular body parts^25,26^. The non-clinical forms are considered to be more prevalent than the clinical manifestation^25,26^. Though it was suggested that animal species, in which colonisation with the fungus is common (e.g. pigeons and koalas), may be responsible for a spread or sustainment of CNGSC strains in the environment^15,27^, the role of animals as sentinels signalling the presence of this pathogen in the environment has also been documented^28,29^. Thus monitoring of both, clinical cases and asymptomatic carriage of *Cryptococcus* among companion and pet animals may be useful for the assessment of the presence of these yeasts in the human environment^25,26,29,30^.

The aim of this study was to assess the CNGSC distribution and population structure of the strains isolated from different types of animal specimens collected in the territory of Poland.

## Results

### Isolation results and population tested

Out of 421 sampled animals, 10 (2.375%) were positive. Strains representing CNGSC were isolated from 13 (1.997%) of the collected samples. Detailed isolation data are given in Table 1. Four animals from the tested group were the source of multiple strains (presenting different colony morphology and/or various melanisation patterns) whereas in the other six, single isolates were detected. The multiple strains represented different major molecular types (the strains number n = 3) and sequence types (n = 3; data not shown). In one case isolates obtained from the same specimen were phenotypically different though represented the same sequence type.

**Table 1:** The results of isolation of CNGSC strains from animal originating specimens.

Animals	Source of specimen	Number of animals	Number and percent of positive animals	Number of samples	Number and percent of positive samples	Number of strains
Feral pigeons (*Columba livia*)	Faeces	130	2 (1.54)	130	2 (1.538)	2
	Swabs*	8	0 (0)	24	0 (0)	0
Domestic pigeons (*Columba livia* f. domestica)	Swabs*	107	7 (6.542)	321	10 (3.115)	13
Parrots (*Nymphicus* sp., *Psittacus* sp.)	Faeces	2	0 (0)	2	0 (0)	0
Capercaillie (*Tetrao* sp.)	Faeces	28	0 (0)	28	0 (0)	0
Chicken (*Gallus* sp.)	Faeces	16	0 (0)	16	0 (0)	0
Red ruffled lemur (*Varecia* sp.)	Throat swab	8	1 (12.5)	8	1 (12.5)	2
Cheetah (*Acinonyx* sp.)	Throat swab	1	0 (0)	1	0 (0)	0
Cat (*Felis catus)*	Nasal swab	53	0 (0)	53	0 (0)	0
Dog (*Canis familiaris*)	Nasal swab	68	0 (0)	68	0 (0)	0
Total		421	10 (2.375)	651	13 (1.997)	17

*Sets of swabs from oropharynx, crop and cloaca taken from every examined bird.

In total, seventeen strains were obtained from the tested specimens, all them were exclusively *C. neoformans*.

Fifteen of the 17 isolates were obtained from 12 (2.53%) positive samples collected from pigeons. Analysing the results of isolation using a swabbing technique in this population, in domestic pigeons *C. neoformans* was detected in 3.115%, whereas it could not be cultured from any of 24 samples collected from the feral ones. Two of 130 samples of feral pigeons’ excreta 2 (1.538%) were positive.

*Cryptococcus neoformans* was also detected in the throat of one of 8 (12.5%) lemurs examined. The results of isolation were negative with respect to other animals tested.

Taking into account the isolates obtained from the tested specimen in our laboratory (n = 17) and those contributed by our partner laboratory (n = 22), a population consisting of 39 strains has been collected, that was used for further analysis. The origin and characteristic of all 39 strains are given in Table 2.

**Table 2:** The results of *URA*5-RFLP, sero/mating type and MALDI-TOF MS analyses.

Strain number	The specimen source	MMT	ST/MT	MALDI-TOF MS		Deposition number
				Score value	MMT recognition	
LRKr	Lemur, throat*	VNIV	Dα	2.155	*C. neoformans* var. *neoformans*	PCM 3109
LRB	Lemur, throat*	VNI	Aα	2.321	*C. neoformans* var. *grubii*	PCM 3110
VL1	Parrot, cloaca	VNI	Aα	2.147	*C. neoformans* var. *grubii*	PCM 3111
VL2	Peacock, cloaca	VNI	Aα	2.281	*C. neoformans* var. *grubii*	PCM 3112
VL3	Domestic pigeon, droppings	VNIV	Dα	1.962	*C. neoformans* var. *neoformans*	PCM 3113
VL4	Domestic pigeon, droppings	VNIV^1^	Dα	2.083	*C. neoformans* var. *neoformans*	PCM 3114
VL5	Domestic pigeon, droppings	VNIV	Da	2.005	*C. neoformans* var. *neoformans*	PCM 3115
VL7a	Domestic pigeon, droppings^	VNIV	Dα	1.981	*C. neoformans* var. *neoformans*	PCM 3116
VL7b	Domestic pigeon, droppings^	VNIV	Dα	1.984	*C. neoformans* var. *neoformans*	PCM 3117
VL9	Domestic pigeon, droppings	VNIV	Dα	1.866	*C. neoformans* var. *neoformans*	PCM 3118
VL10	Domestic pigeon, droppings	VNIV	Dα	1.945	*C. neoformans* var. *neoformans*	PCM 3119
VL11	Domestic pigeon, droppings	VNIV	Dα	**1.822**	*C. neoformans* var. *grubii*	PCM 3120
VL12	Domestic pigeon, droppings	VNI	Aα	2.234	*C. neoformans* var. *grubii*	PCM 3121
Vl13	Domestic pigeon, droppings	VNIV	Dα	2.027	*C. neoformans* var. *neoformans*	PCM 3122
VL14	Cat, throat	VNIV	Dα	**1.631**	*C. neoformans* var. *neoformans*	PCM 3123
VL15	Domestic pigeon, cloaca	VNIV^1^	Dα	2.057	*C. neoformans* var. *neoformans*	PCM 3124
VL16	Domestic pigeon, throat	VNIV^1^	Dα	2.074	*C. neoformans* var. *neoformans*	PCM 3125
VL17	Domestic pigeon, crop	VNIV	aADa	1.991	*C. neoformans* var. *neoformans*	PCM 3126
VL18	Domestic pigeon, droppings	VNIV	Dα	1.997	*C. neoformans* var. *neoformans*	PCM 3127
VL19	Domestic pigeon, droppings	VNIV	Dα	1.836	*C. neoformans* var. *neoformans*	PCM 3128
VL20a	Domestic pigeon, droppings**	VNIV	Dα	1.774	*C. neoformans* var. *neoformans*	PCM 3129
VL20b	Domestic pigeon, droppings**	VNIV	Dα	1.738	*C. neoformans* var. *neoformans*	PCM 3130
VL21a	Domestic pigeon, droppings”	VNIV	Dα	2.010	*C. neoformans* var. *neoformans*	PCM 3131
VL21b	Domestic pigeon, droppings”	VNIV	Dα	2.101	*C. neoformans* var. *neoformans*	PCM 3132
GKZ1	Domestic pigeon, crop#	VNIV	Dα	2.060	*C. neoformans* var. *neoformans*	PCM 3133
GKZ2	Domestic pigeon, throat #	VNIII	αADα	2.036	AD hybrid	PCM 2996
GKZ3	Domestic pigeon, throat #	VNIV	Dα	2.006	*C. neoformans* var. *neoformans*	PCM 3134
GAW1	Domestic pigeon, crop	VNIV	Dα	2.106	*C. neoformans* var. *neoformans*	PCM 3135
GAW2	Domestic pigeon, cloaca +	VNIV	Dα	2.056	*C. neoformans* var. *neoformans*	PCM 3136
GAW3	Domestic pigeon, crop +	VNIV	Dα	2.018	*C. neoformans* var. *neoformans*	PCM 3137
GAW19	Domestic pigeon, crop	VNIII	αADα	2.150	AD hybrid	PCM 2999
GAW24	Domestic pigeon, crop	VNIV	Dα	1.979	*C. neoformans* var. *neoformans*	PCM 3138
GAW26	Domestic pigeon, throat	VNIV	Dα	2.116	*C. neoformans* var. *neoformans*	PCM 3139
GAW11w1	Domestic pigeon, crop ~	VNIV	Dα	1.801	*C. neoformans* var. *neoformans*	PCM 3140
GAW11w2	Domestic pigeon, crop ~	VNIV	Dα	2.027	*C. neoformans* var. *neoformans*	PCM 3141
GAW11w4	Domestic pigeon, crop ~	VNIII	αADα	2.127	AD hybrid	PCM 2997
GAW11b	Domestic pigeon, throat ~	VNIII	αADα	2.209	AD hybrid	PCM 2998
L1	Feral pigeon, droppings	VNI	Aα	2.231	*C. neoformans* var. *grubii*	PCM 3142
L2	Feral pigeon, droppings	VNI	Aα	2.145	*C. neoformans* var. *grubii*	PCM 3143

In bold—isolates with score value below 1.7 or misidentified on subspecies level by MALDI-TOF MS.

VL—strains obtained from Veterinary Laboratory Vetlab.

VNIV^1^—VNIV strains presenting an atypical *URA*5-RFLP banding pattern. MMT major molecular type; according to *URA*5-RFLP. ST/MT sero- and mating type. AD hybrid = *C. neoformans* var. *grubii* x *C. neoformans* var. *neoformans*. PCM—Polish collection of microorganisms.

*,^,#, ~ , + ,**,“– multiple isolates obtained from the same animal.

### Analysis of the MMT, sero- and mating type

The most frequently isolated MMT was VNIV with 29 strains (74.36%,) followed by VNI (6; 15.38%) and VNIII/AD hybrid (4; 10.26%). Five of the VNIV strains (12.82%), presented an atypical *URA*5-RFLP banding pattern described by Florek et al.^31^ These along with four VNIII isolates bearing the same *URA*5-AT32 allele, represented 23.07% of the whole strain population described in the present study. With respect to sero- and mating type evaluation, among the haplotype strains 27 were classified as the type Dα, six as Aα and one as Da. The group of AD hybrids presented the type αADα. Interestingly, one of the strains (VL17) recognised by *URA*5-RFLP method as VNIV, in the sero- and mating-type assessments was assigned as aADa. Detailed information concerning results of *URA*5-RFLP analysis and determination of sero- and mating-type are given in Table 2.

### MALDI-TOF MS analysis

Using Biotyper’s 3.1 software library database and the manufacturer’s score values threshold, 38 of 39 strains (97.44%) were correctly identified achieving a minimal score above 1.7. Fifteen isolates (38.46%) reached a score value range 1.7–1.99. With respect to the other 22 (56.41%) isolates, identification of secure genus/probable species level was possible (the score range 2.00–2.299). Only in one (2.56%) case, was the score value secured a highly probable species recognition (the score above 2.3). Misidentifications were not observed after taking the best result (score value) out of one to six analyses performed for every strain. An upgrade of the database, consisting of the addition of the spectra of eight CBS reference strains, caused only subtle shifts among the above described groups. The number of the correctly identified strains, as well as isolates with a score value above 2.3, remained the same. Thirteen (33.33%) strains were assigned as probable genus, while 24 (61.54%) as secure genus/probable species. Further extension of the database, with the spectra of genetically recognised strains isolated in Poland, influenced the score of only one strain tested.

Taking into consideration sub-species/major molecular type level identification, the manufacturer’s database alone enabled correct recognition of all VNI and all but one VNIV strains, by analysis of best/first match strains. The one miss-matched VNIV isolate was paired with VNI P152 CBS strain and in-house obtained reference spectra did not improve the recognition. As the VNIII standard was absent in the original database, none of the examined four AD hybrids were identified correctly on the sub-species level. These hybrids were linked by Biotyper’s software with both parental MMTs, and one of them with the VNI/VNII hybrid. Application of the extended database containing strain CBS 132, enabled correct identification of 100% of the VNIII strains. At the same time, scores obtained by all the hybrids have increased, enabling more secure identification according to the manufacturer’s score values threshold system. The score values and MMT identification results obtained by this method are given in Table 2.

### Susceptibility to antifungals

The activities of amphotericin B, 5-fluorocytosine and triazole derivatives against the herein isolated, animal CNGSC isolates are presented in Table 3.

**Table 3:** Distributions of MIC of amphotericin B, 5-fluorocytosine and triazole derivatives for animal isolates of *Cryptococcus neoformans* species complex.

Antifungal agent	MMT (N)	Number of isolates with the MIC (mg/L):													MIC50	MIC90	non-WT
		0.015	0.03	0.06	0.125	0.25	0.5	1	2	4	8	16	32	> 64			
Amphotericin B	VNI (6)^1^						1	5									
	VNIII (4)						1	3									
	VNIV (29)^1^				6	1	12	10							0.5	1	
	Total (39)				6	1	14	18							0.5	1	
5-Fluorocytosine	VNI (6)										2	3	1				
	VNIII (4)										1	2	1				
	VNIV (29)							1	3	3	9	5	4	4	8	32	4
	Tolal (39)							1	3	3	12	10	6	4	8	32	4
Fluconazole	VNI (6)^2,3^					1					1	3	1				
	VNIII (4)^2^					1	1	2									
	VNIV (29)^3^			1		1	4	7	3	9	4				2	8	
	Total (39)			1		3	5	9	3	9	5	3	1		2	8	
Itraconazole	VNI (6)		3	1	1	1											
	VNIII (4)		3		1												
	VNIV (29)	2	11	2	3	7	4								0.125	0.25	
	Total (39)	2	17	3	5	8	4								0.06	0.25	
Isavuconazole	VNI (6)	4	1		1												
	VNIII (4)	1	3														
	VNIV (29)	7	3	14	5										0.125	0.125	
	Total (39)	12	7	14	6										0.03	0.125	
Posaconazole	VNI (6)	3		2		1											
	VNIII (4)		2		1	1											
	VNIV (29)	6	2	6	5	9	1								0.125	0.25	
	Total (39)	9	4	8	6	11	1								0.06	0.25	
Voriconazole	VNI (6)			3	3												
	VNIII (4)			1	3												
	VNIV (29)	2	6	12	7	1									0.06	0.125	
	Total (39)	2	6	16	13	1									0.06	0.125	

MIC90—the minimal concentration of the drug with is able to inhibit the growth of 90% strains of tested population.

MIC50—the minimal concentration of the drug with is able to inhibit the growth of 50% strains of tested population.

^1^p = 0.028 (VNIV vs. VNI).

^2^p = 0.003 (VNI vs. VNIII).

^3^p = 0.019 (VNI vs. VNIV).

The obtained MICs of amphotericin B did not exceed 1 mg/L (range 0.125–1 mg/L) and according to EUCAST recommendations, all the tested isolates were classified as susceptible to this drug. The observed MICs of 5-fluorocytosine ranged from 1 to > 64 mg/L. Four isolates (10.26% of the group tested) presenting the highest MIC (> 64 mg/L) were listed to the non-WT population. All of the non-WT isolates represented MMT VNIV. The MIC values established for fluconazole ranged from 0.06 to 32 mg/L, with MIC_50_ and MIC_90_ being 2 and 8 mg/L, respectively. MICs for itraconazole, isavuconazole, voriconazole, and posaconazole ranged from 0.015 mg/L to 0.5, 0.125, 0.25, and 0.5 mg/L, respectively. The MIC_50_ and MIC_90_ concerning the triazoles tested were as follows 0.06 mg/L and 0.25 mg/L (itraconazole), 0.03 mg/L and 0.125 mg/L (isavuconazole), 0.06 mg/L and 0.125 mg/L (voriconazole), and 0.06 mg/L and 0.25 mg/L (posaconazole). According to the ECVs adopted, phenotypes other than WT were not found with regard to any of the tested triazoles.

Comparing the susceptibility of the MMTs, statistically significant differences were found between MMT VNIV and VNI in the MIC values of amphotericin B (p = 0.028) as well as in the MICs of fluconazole between MMT VNI vs. VNIII (p = 0.003), and VNI vs. VNIV (p = 0.019).

## Discussion

Due to its important role as a human and animal pathogen, the CNGSC has been extensively studied, especially with regard to its distribution in the environment and genetic structure of both clinical and environmental populations. In Poland however, this field of research has been barely touched^32–35^. The only available study considering genetic structure of *C. neoformans* strains isolated in Poland was performed during the European Confederation of Medical Mycology (ECMM) prospective survey of cryptococcosis in Europe^19^, where 7 Polish clinical strains MT-VNI were contributed. Similarly, data concerning prevalence of the CNGSC in animals in our country is almost absent^36^.

In our investigation, specimens obtained from 421 animals representing both birds and mammals were analysed.

Animals can be considered sentinel hosts for pathogens causing different human diseases, among them are those like cryptococcosis, caused by environmentally acquired microorganisms. Taking into account behavioural characteristics, animals are more prone than humans to get contaminated with or infected by cryptococci^23,24,37^. Subclinical carriage of *Cryptococcus* species in certain animal sites (e.g. mucous membrane of the nasal cavity, oropharynx, crop, cloaca as well as, to some degree, skin) has been widely documented^25–28,30^. Sampling of these sites proved to be useful for detection of the pathogen even in situations, in which finding of the primary environmental sources was impossible^28,38^. Processes such as natural air filtration in the nasal cavity leading to a concentration of pathogens particles, make animals amplifiers and ideal environmental samplers^26,28^. Several groups of animals were indicated as sentinels of the environmental CNGSC presence: companion and pet animals, animals for which trees stand for a natural habitat or a food source, rodents living in the proximity of households and birds^21,25,28,38,39^.

In this study, 10 of 421 tested animals (2.37%) and 16 of 651 (1.997%) tested samples of animal origin were positive for *C. neoformans*. To our knowledge not many researchers have conducted an analysis of this yeast prevalence in multi-species, non-clinical animal groups. The results presented in Montagna et al.^40^, analysing 758 samples collected in Italy from birds and mammals showed the incidence of the fungus to be higher, amounting to 5.1%.

The role of birds, especially pigeons as a source or mechanic carriers/spreaders of *C. neoformans* has been widely discussed^15,41–43^, thus prevalence of the pathogen in pigeons has been tested worldwide. In our investigation, the overall incidence rate of the yeast in pigeon specimens was 2.737%, and similarly to other authors’ observations^44–46^, the fungus strains were isolated more often in domestic birds, when compared to the feral variety.

With regard to pigeons and the most readily tested specimen obtained from those birds, which is excreta, it was proved that the *Cryptococcus* isolation rate was strongly correlated to the specimen composition as well as to local physical conditions like temperature, humidity, direct access to sunlight and hygiene of the cages^15,47,48^. Even the status of the specimen (e.g. fresh vs. dry) influenced this isolation ratio in a statistically important manner^47,49^. It is worth mentioning that major local fluctuations concerning the isolation ratio were reported, with respect to country regions or even districts of the same city^46,47^. Notable differences among studies were noticed in regard to the prevalence of *C. neoformans* in pigeon faeces. In Europe, negative results of the isolation were observed in Sweden, Austria and Germany^50,51^, whereas in countries characterised by a warmer climate like Italy, Portugal and Spain, those numbers were far higher, reaching even over 60%^45,46,52^. In Russia, the ratio amounted to 3.2%^18^ that was only slightly higher than results of our work.

It was shown^53^ that after an experimental pigeon crop inoculation with *C. neoformans*, the pathogen was present in faeces as fast as one hour after the procedure, and was continuously excreted up to 24 h. This investigation has proved that the passage of the fungus through pigeon gastrointestinal tract is possible. This observation together with the fact birds’ feeding habits result in constant exposure of its gastrointestinal tract to the microorganism, suggest that oropharynx, crop and cloaca may prove a valuable specimen source in the search for *C. neoformans* environmental presence. In our studies, 345 swabs were taken from the oropharynx, crop and cloaca of 8 feral and 107 domestic pigeons. Of these obtained from feral ones, none was positive. However, singular or multiple colonies of the fungus were isolated from the swabs obtained from 7 domestic birds. The pathogen was present in 3.48% of oropharyngeal samples, 5.21% of those taken from the crop and 0.87% of cloacal samples. In the studies conducted by other authors, isolation ratio with respect to crop material varied substantially, amounting to 1.1 to 9.6%^54,55^. Examination of the cloacal specimen, in some cases^56^ gave negative results, whereas in other^57^ this fungus was present in 1.81% of the tested samples, similarly to our findings. To the best of our knowledge, data considering contamination of pigeons’ oropharynx was not available to date.

One hundred and twenty one nasal swabs were obtained from 53 cats and 68 dogs in this study. The samples were collected using superficial or deep swabbing techniques. Interestingly, fungi belonging to CNGC were not detected in any of the examined swabs. A similar isolation result was observed in Russia^18^, where 47 both canine or feline samples gave negative cultures. The incidence of contamination or colonization of nasal passages with CNGSC in cats and dogs has been studied in Australia, Canada and Italy. It was observed that the isolation rate concerning the fungus may be influenced by such conditions as local exposition, animal species and origin (domestic vs. feral) and sampling technique. Therefore, the prevalence observed by other authors ranged from 1.56% to 15% with respect to feline and from 10.7% to 14% with respect to canine specimens^25,26,30^.

The survey of cryptococcosis performed under the umbrella of the ECMM, was one of the first research studies that greatly contributed towards the understanding of *C. neoformans* population structure in Europe^19^. Results of the survey showed that among analysed clinical strains, the dominant group was serotype A. It was also observed, however, that in Europe, serotypes D and AD were often isolated from the patients. Analysis of both clinical and environmental/veterinary strains’ data, performed by Cogliati^58^, confirmed the previous observation concerning the higher occurrence of the MMT VNIII and VNIV strains (18.5% each MMT) in Europe. An environmental survey performed by the ISHAM’s Working Group^15^, revealed that within an arboreal specimen, collected in western and southern Europe, as well as in non-European countries of the Mediterranean region, strains of the MMT VNI were identified in 69.62%, VNII in 0.42%, VNIII in 7.38% and VNIV in 22.57% of *C. neoformans* isolates. Interestingly, the prevalence of particular MMTs was geographically highly variable. Examination of the genetic structure of Polish, herein presented *C. neoformans* strains demonstrated, the population consisted mainly of MMT VNIV (74.36%) and to a lesser degree both MMT VNI (15.38%) and MMT VNIII (10.26%). Several observations have been made associating better tolerance for low temperatures and higher prevalence of *C. neoformans* var. *neoformans* in the environment of certain geographical regions, compared to other members of the CNGSC^59,60^. This phenomenon may explain the here documented predominance of the VNIV strains observed within the analysed population.

In the context of the genetic structure of the *C. neoformans* population isolated from animals in Europe, the available data is widely varied. Whereas some populations consisted exclusively or almost exclusively of MMT VNI^40,46^, in others an equal distribution of MMT VNI and VNIV^30^ or even predomination of MMT VNIV (65.2%) were observed^45^. Neither geographical nor animal origin of the strains analysed, seemed to influence those population structures in an important manner. Another MMT observed in higher numbers in Europe, the VNIII, was isolated in studies concerning an animal specimen in percentages ranging from 0 to 17.4^30,45^. In line with our data, among European animal strains, those representing MMT VNII were not detected, with the exception of the VNII/VNIV hybrid described by Danesi et al.^30^.

As an extension of the genetic structure analysis performed in the present study, the distribution of sero- and mating types among *C. neoformans* strains was examined. All the isolates obtained in our research belonging to the serotype A or AD as well as most (27 of 29) of those representing serotype D, were characterised by the presence of *MAT*α locus. The group of AD hybrids (MT-VNIII according to *URA*5-RFLP) was αADα. The *MAT*a locus was detected in one of the serotype D isolates. This locus was also observed in the strain, which according to the *URA5*-RFLP method was assigned to MMT VNIV, while sero- and mating type analysis classified it as the aADa hybrid. The studies performed by different authors in Europe, concerning both clinical and environmental strains, showed a predomination of *MAT*α locus, which was present in the vast majority of the serotype A isolates (97.9 to 99.3%) and in most of the serotype D ones (73 to 95.6%)^15,19^. Among hybrids investigated in those works types αADα, aADα, αADa, Aα and Dα were observed. Investigations on animal strains isolated in Europe^30,40,46,61^, confirmed the results of the above-mentioned works as well as of our studies, indicating that among European isolates MATα-bearers dominate, regardless of the MMT/serotype. With respect to *MAT*a, presence of this locus is rare and it is more commonly observed in the VNIV strains. It was suggested^62^ that the higher prevalence of the *MAT*a allele in the MMT VNIV strains may be responsible for more frequent sexual reproduction, than that observed in the sibling variety, leading to a higher variability as a result of the recombination.

MALDI-TOF MS is considered to be cost and time effective as well as a reliable diagnostic tool widely used by clinical and experimental microbiologists. The method enables rapid and reproducible identification and can be applied to a vast range of microorganisms. Though several authors reported the usefulness of this technique in the recognition of CNGSC at the species and even sub-species level, it was also observed that the presence of the capsule as well as the cell wall composition, make MALDI-TOF MS identification of these pathogens challenging in comparison to other clinically important fungal agents^63,64^. Additionally, it was noticed that in some of the manufacturer’s library versions, spectra of particular types belonging to the CNGSC were underrepresented, therefore construction of the in-house libraries were beneficial for the increase in identification quality^65,66^.

Correct identification percentage values obtained by authors studying the application of this identification method with regard to *Cryptococcus* ranged from 0 to 100%^64–68^. The differences in the observed values depended mainly on the strain tested (e.g. its MMT etc.), the type of spectrometer and platform used, protein extraction protocol, version of the database applied and in-house database supplementation, as well as threshold value accepted for the study^68–72^. Several authors also indicated the role of the media used for strains cultivation^64,68^ or usage of strains obtained from culture collections versus fresh isolates^64^. In our study, correct identification, which was defined as recognition in accordance with the results of other methods used and score value ≥ 1.7, was achieved for 97.4% of the strains, regardless of whether or not in-house library supplementation was applied. The result seems similar to those obtained in other investigations, where use of MALDI-TOF MS made it possible to correctly recognise 97.14 to 100% of the strains tested^66,70,73,74^. There is a group of publications presenting lower results for the method with respect to *C. neoformans* (62.5–87.5%) but it may be the outcome of a small number of the tested isolates (3 to 8 strains)^63,68,74^.

The application of MALDI-TOF MS for identification of the CNGSC strains on the sub-species (MMT) level has been reported^65,66^. The largest in-house library constructed to date, containing spectra of 160 strains, enabled the correct assignment of all the tested isolates representing haploid molecular types as well as intra-species hybrids. But not all inter-species hybrids were recognised correctly^66,75^. In our research it was possible to recognise MMT in 34 of 35 haploid strains, with application of the manufacturer’s database alone. The misrecognised strain belonged to MMT VNIV. During analysis of AD hybrids in our study, addition of only one AD reference strain to the original library has improved the sub-species level recognition rate from 0 to 100%. Difficulties with regard to correct recognition of the MMT VNIV and AD hybrids strains, even after supplementation of the manufacturer’s libraries with in-house created reference spectra, were also reported by the others^3,73^. It might suggest that the number of reference spectra of the problematic MMTs is still underrepresented when compared to the remaining ones. Available data as well as results of our study confirm that adopting certain protocols, MALDI-TOF MS provides rapid and reliable identification on a species and subspecies level that is of great importance, especially when it concerns CNGSC infections.

The characteristics of the *C. neoformans* strains isolated in the present study was supplemented by the examination of their susceptibility to antifungal drugs. Use of the reference (microdilution) method made it possible to obtain unambiguous results, expressed as minimal inhibitory concentration (MIC) of the drug, however an assessment of the strains as susceptible or resistant was difficult due to problems with establishing the interpretative criteria. Based on the available literature, it was observed that except for amphotericin B, posaconazole and voriconazole, for which clinical breakpoints or ECVs were established with regard to *C. neoformans*, published data concerning other antifungals is often inconsistent. Different results obtained by the researchers, could be related to technical approaches (e.g., applying of CLSI or EUCAST methodology) or ECV definition (e.g., ECV 90 or 97%) as well as characteristics of the tested population of isolates (size, genotypic diversity, geographical and ecological origin)^76,77^. The described discrepancies are of great importance especially with respect to 5-fluorocytosine, which is a drug recommended in the therapy of cryptococcal meningitis^78^. In the present study, for interpretation of the results obtained for 5-fluorocytosine, ECV of 32 mg/L proposed by Córdoba et al.^76^ was applied, enabling detection of four non-WT isolates (MIC > 64 mg/L), possible holders of acquired resistance mechanisms. On the contrary, Espinel-Ingroff et al.^79^ have demonstrated separate ECVs for MMT VNI and for non-typed *C. neoformans* (8 mg/L and 16 mg/L, respectively). Taking into account ECVs obtained by the latter authors, the category non-WT should pertain to four out of six analysed in our study VNI isolates or even 10/39 representatives of all the population (regardless of the genotype). This data indicates that at least 10% of the environmental isolates obtained in this study may be holders of resistance to 5-fluorocytosine and in case of infection may not respond to this therapy. The percentage obtained in our study is much higher than the rate of 5-fluorocytosine-resistant isolates found by other authors, where strains with MIC higher than 32 mg/L usually constituted between 1 and 2.5% of the population^76,79–81^. Substantial differences can also be found among ECVs reported for triazole derivatives. As an example, these values for fluconazole were established as 32, 16 or 8 mg/L^76,77,81^ that means if applied in our studies, 0, 2 or 4 non-WT isolates respectively would be detected. The prevalence of fluconazole-resistance in clinical isolates of *Cryptococcus* spp. was recently evaluated by Bongomin et al.^82^ The authors analysed susceptibility of almost 5000 *Cryptococcus* isolates described in 29 papers from 5 continents. The rate of fluconazole–resistance, detected in these studies ranged from 0 to 50%. The mean was 10.6% for isolates from the first incident, and 24% for those originating from relapse cases. The cited data indicated that fluconazole–resistance usually develops secondarily to therapy with this drug. The animal isolates tested in our study were not exposed to fluconazole and other triazoles, and probably for this reason their triazole-susceptibility was that high.

Some authors demonstrated that MMTs of CNGSC could differ significantly in their antifungal susceptibility. As an example, Lee et al.^83^, who studied Australian *Cryptococcus* collection found mean MIC values of 5-fluorocytosine significantly higher for *C. neoformans* VNI, than those for *C. gattii* VGI (p = 0.002), as well as mean MICs against fluconazole for VNI significantly higher than those for VNII (p = 0.036). Similarly, in our study some significant differences between MMTs were also depicted. The average MIC values of amphotericin B were lower (p = 0.028) and of fluconazole were higher (p = 0.019) for VNIV than for VNI. The values for the fluconazole were also higher when comparing MMT VNI and VNIII.

*In conclusion*, our study presented for the first time prevalence and structure of non-clinical CNGSC in Poland. In the analysed population of the strains of animal origin, isolates belonging to the MMT VNIV were present at a higher percentage rate, than that observed to date in Europe. Despite the fact, that sampled animals were not reported as cryptococcosis cases and subsequently treated with antifungals, some degree of resistance to 5-fluorocytosine has been detected, indicating the existence of the resistant strains in the environment. Further studies, concerning arboreal strains of the Polish CNGSC, are required in order to establish whether examination of the strains obtained from sentinel animals alone is adequate to characterise the whole population of the environmental strains or if animal/arboreal factors may somehow alter its structure.

## Material and methods

### Specimen and isolates

A total of 651 samples were collected from 421 animals between August 2015 and July 2019. Most of them were obtained in the territory of Lower Silesia (Wrocław or its surroundings, n = 612), yet several represented different areas of Poland (n = 39). Detailed information related to bird and mammalian specimen collection is available in Table 1.

Out of the all the samples, 475 specimens were swabs taken from the oropharynx (n = 124), crop (n = 115), cloaca (n = 115) and nasal cavity (n = 121), whereas the remaining 176 were excreta. Swabs obtained directly from pigeons (both domestic and feral) were collected during the annual routine clinical examinations of health status in pigeon flocks. Faecal samples obtained from feral pigeons were collected from the surroundings of public buildings, pavements and streets. Samples originating from other birds’ species (chicken, Capercaillie) were provided to us by private owners, or centres for wild game breeding. Nasal swabs were obtained from our Faculty’s cat and dog patients and submitted to our laboratory in order to perform microbiological investigation. Similarly, throat swabs from lemurs and cheetah were submitted to our laboratory by zoo veterinarians (Zoological Garden, Opole). The methods of the specimens collecting were carried out in accordance with article 37ah–37ak of the Pharmaceutical Law Act (Dz. U. z 2019 r. poz. 499, 399 i 959 [Journal of Laws of the Republic of Poland from 2019, item. 499,399 and 959]) from September 6th 2001, and according to the Experiments on Animals Act (Dz. U. 2019 poz. 1392 [Journal of Laws of the Republic of Poland 2019, item. 1392]) from July 5th 2019, the approval of the Ethical Committee was not required. The informed consent was obtained from the owners of all animals.

Faecal samples (1 g) were vortexed with 20 mL of a sterile saline solution and left in order to let the suspension settle. Then supernatants were diluted 1:10. Two sets of plates containing Niger seed agar (NSA) were inoculated with 100 µL of the supernatant and its dilution, respectively. Swabs taken from animals were inoculated directly onto NSA plates. The plates were incubated at 30 °C for up to 14 days, though positive samples could usually be detected at 48–96 h. All brown colonies cultured from each sample were subcultured as single-colony isolates on NSA in order to purify cultures, and then assessed morphologically using India Ink staining. Strains producing melanin on NSA and positive in morphological evaluation were classified as CNGSC. A selection of isolates grown from one sample for further tests was performed by means of the colony morphology and melanisation pattern analysis.

Aside from the presented above specimen, 22 biochemically identified *C. neoformans* isolates of animal origin, obtained from the commercial veterinary laboratory (Vetlab, Wrocław, Poland) were included in our study. These strains originated from the Lower Silesia territory (n = 6) or from other parts of Poland (n = 13).

### DNA isolation and PCR

The CNGSC strains were cultured on Sabouraud dextrose agar (SDA) for 48 h at 30 °C. DNA extraction was performed using the MasterPure Yeast DNA Purification Kit (Epicentre Biotechnologies, Madison, WI, USA) according to the manufacturer’s instructions. All PCRs presented in this study were performed in MJ Mini Personal Thermal Cycler (BIO-RAD, Hercules, USA) in 25 µL reaction volume consisting of 1 µL of the extracted DNA, 12.5 µL of master mix (*Taq* DNA polymerase 0.05 U/µL, MgCl_2_ 2 mM, dNTP-mixed solution 4 × 0.25 mM, a loading buffer and a red stain (PCR Mix, A&A Biotechnology, Gdynia, Poland)), 20 pM of each primer and 11.1 µL of water. Sterile water was used as negative control in each assay.

### Sequencing of the *SOD*1 gene

In order to recognise species and/or variety, sequencing of the *SOD*1 gene was performed^84^. For amplification of the gene in *C. gattii* and *C. neoformans* var. *grubii* (MMT VNI), primers presented in the MLST consensus scheme were used^2^. For *C. neoformans* var*. neoformans* (MMT VNIV) an alternative reverse primer for the *SOD*1 gene described by Sanchini et al.^85^ was applied. Obtained PCR products were purified and sequenced (DYEnamic ET Terminator Cycle Sequencing Kit, Amersham Biosciences Europe GmbH, Germany). Forward and reverse sequences were assembled using BioEdit v7.2.0 (http://www.mbio.ncsu.edu/BioEdit/bioedit.html) and then analysed by BioloMICS Polyphasic Identification Software (http://mlst.mycologylab.org/cneoformans). The recognition was obtained using the Fungal MLST database for *C. neoformans* and *C. gattii.* Sequences representing potentially new alleles were submitted to this database in order to confirmed their novelty.

### Sero- and mating type determination

 of the tested strains were established using the PCR-based method of amplification of the serotype-specific and mating-type-specific *STE*20 gene, described by Li et al.^86^ The following strains, representing particular sero- and mating types, were used as positive controls: CBS 10084 (Aα), CBS 132 (AαDa), IUM 96–2828 (Aa) and CBS 10079 (Dα).

### Restriction fragment length polymorphism analysis of the orotidine monophosphate pyrophosphorylase gene (*URA*5-RFLP)

The *URA5*-RFLP technique was conducted according to Meyer et al.^87^ PCR products were double digested with *Cfr*13I (*Sau*96I) and *Hha*I enzymes (Thermo Fisher Scientific, Waltham, MA, USA) for 16 h and separated in 3% agarose gel at 100 V for 3 h. RFLP patterns of the tested strains were determined visually by comparison with standard strains representing major molecular types (CBS 8710-VNI, CBS 10084-VNII, CBS 132-VNIII and CBS 10079-VNIV).

### Matrix Assisted Laser Desorption/Ionization Time-of-Flight Mass Spectrometry (MALDI-TOF MS) Analysis

 was performed as described by Król et al.^88^, with modifications consisting of cultivation conditions and extraction procedure applied. For the extraction, content of two to five colonies of freshly grown cultures, incubated on SDA at 30 °C for 48 h, was suspended in 300 µL of double-distilled water. Subsequently, 900 µL of absolute ethanol were added. The sample was centrifuged (13,000×*g* for 3 min) two times and the sediment was dried at room temperature. Lysates were obtained by adding 50 µL of 70% formic acid to the pellet, then after mixing thoroughly, 50 µL acetonitrile was added and the sample was mixed again. Following centrifugation (13000×*g* for 2 min), the supernatant was placed into a fresh tube, and 1 µL of the protein lysate was applied on a 384 ground steel MALDI target plate (Bruker Daltonics, Bremen, Germany) and air-dried at room temperature. Next the sample was coated with 1 µL of α-cyano-4-hydroxycinnamic acid matrix solution (HCCA; Bruker Daltonics) and air-dried again. Biotyper 3.1 software (Bruker Daltonics) and a database that contains 6904 strain entries, were used for the identification. According to the manufacturer instructions, the given score values were used: < 1.7 (identification not reliable), 1.7–2.0 (probable genus identification), 2.0–2.3 (secure genus identification and probable species identification), and > 2.3 (highly probable species identification). The highest scores obtained in series of repetitions were given as the result. In order to enable sub-species (major molecular type) level identification, spectra of the strains representing particular MMTs were added to the Biotyper database. For the first step of database upgrading, reference strains corresponding with ISHAM MLST consensus scheme, originally not included in the database were added, namely CBS 6289, CBS 10101, CBS 10082, CBS 10081, CBS 10084, CBS 8710 and CBS 132 (Westerdijk Fungal Biodiversity Institute, Utrecht, the Netherlands). For the second step of the database extension, five selected strains isolated in our study and analysed using MLST (data not shown) and *URA*5-RFLP methods (LRB-VNI, SK4-VNI, 204-VNII, GAW19W-VNIII, and 1°-VNIV) were introduced. For each of the reference strains, 24 replicates of sample were analysed by MALDI-TOF MS. The spectra obtained were analysed by Flex Analysis software (version 3.4) and single spectra with peaks differing from the others and with small intensity were removed, resulting in at least 20 good quality spectra. These selected spectra were used to create a reference Main Spectrum Profile (MSP) using Bruker Biotyper 3.1 software. The obtained MSP were implemented to MALDI-TOF Biotyper database. In order to define the molecular type of the examined isolates, the best match strains’ MMTs were analysed.

### Susceptibility to antifungals

The susceptibility of CNGSC isolates to amphotericin B, 5-fluorocytosine, fluconazole, isavuconazole, itraconazole, posaconazole, and voriconazole was examined with the microdilution method according to the European Committee on Antimicrobial Susceptibility Testing (EUCAST) Definitive Document E.DEF 7.3.1^89^. All used reagents were obtained from Sigma-Aldrich Life Science.

The minimal inhibitory concentration (MIC) was defined as the lowest drug concentration resulting in 90% (amphotericin B) or 50% (5-fluorocytosine and triazole derivatives) reduction of the OD530, when compared to the drug-free control. The strains *Candida parapsilosis* ATCC 22019 and *C. krusei* ATCC 6258 were used as a quality control.

The clinical breakpoints for amphotericin B (1 mg/L) as well as epidemiological cut-off values (ECVs) for posaconazole (0.5 mg/L) and voriconazole (0.5 mg/L) established by EUCAST for *C. neoformans*^90^ were applied. Taking into account the lack of the same criteria for other antimycotics, it was decided to use ECVs published by other authors (32 mg/L for 5-fluorocytosine and fluconazole, 0.5 mg/L for itraconazole and 0.125 mg/L for isavuconazole)^76,77,91^. While according to the clinical breakpoints, strains were identified as susceptible or resistant, the used ECVs enabled categorization of isolates into wild-type (WT; population of isolates in a species-drug combination with no detectable acquired resistance mechanisms^77^) or non-wild-type (non-WT; mutation holders).

In order to compare the distribution of MICs between particular MMTs, the Kruskal–Wallis test with post-hoc Duncan method was applied using PAST for Mac OS X v.4.0 (Øyvind Hammer 1999–2020) software. In each analysis a significance level of 5% was adopted.

## Data Availability

All data generated or analysed during this study are included in this published article. Sequences representing possibly new alleles of the *SOD*1 gene were deposited into Fungal MLST database for *C. neoformans* and *C. gattii.* The strains used in the present study were deposited into Polish Collection of Microorganisms (PCM) at Hirszfeld Institute of Immunology and Experimental Therapy PAS. The deposit numbers are given in Table 2.
